# SHIMS 3.0: Highly efficient single-haplotype iterative mapping and sequencing using ultra-long nanopore reads

**DOI:** 10.1371/journal.pone.0269692

**Published:** 2022-06-14

**Authors:** Daniel W. Bellott, Ting-Jan Cho, Emily K. Jackson, Helen Skaletsky, Jennifer F. Hughes, David C. Page

**Affiliations:** 1 Whitehead Institute, Cambridge, Massachusetts, United States of America; 2 Department of Biology, Massachusetts Institute of Technology, Cambridge, Massachusetts, United States of America; 3 Howard Hughes Medical Institute, Whitehead Institute, Cambridge, Massachusetts, United States of America; USDA Agricultural Research Service, UNITED STATES

## Abstract

The reference sequence of structurally complex regions can only be obtained through a highly accurate clone-based approach that we call Single-Haplotype Iterative Mapping and Sequencing (SHIMS). In recent years, improvements to SHIMS have reduced the cost and time required by two orders of magnitude, but internally repetitive clones still require extensive manual effort to transform draft assemblies into reference-quality finished sequences. Here we describe SHIMS 3.0, using ultra-long nanopore reads to augment the Illumina data from SHIMS 2.0 assemblies and resolve internally repetitive structures. This greatly minimizes the need for manual finishing of Illumina-based draft assemblies, allowing a small team with no prior finishing experience to sequence challenging targets with high accuracy. This protocol proceeds from clone-picking to finished assemblies in 2 weeks for about $80 (USD) per clone. We recently used this protocol to produce reference sequence of structurally complex palindromes on chimpanzee and rhesus macaque X chromosomes. Our protocol provides access to structurally complex regions that would otherwise be inaccessible from whole-genome shotgun data or require an impractical amount of manual effort to generate an accurate assembly.

## Introduction

### Background and applications

Reference genome sequence quality is of central importance to modern biological research. Experiments based on aligning cheap and abundant short reads to existing reference sequences have become commonplace, permitting studies of variation by genome and exome resequencing, transcription by RNA sequencing, and epigenetic modifications by chromatin immunoprecipitation–sequencing. However, these experiments are limited by the quality and completeness of the underlying reference sequence, so that new insights may emerge from reanalyzing short-read datasets in the light of an improved reference sequence. The foremost obstacles to accurate reference genome assembly are repeated sequences within the genome. The most structurally complex repeats are ampliconic sequences–euchromatic repeats with greater than 99% identity over more than 10 kb [1]. The complex repetitive structures in amplicons mediate deletions, duplications, and inversions associated with human disease [2]. Amplicons pose special challenges for genome assembly, requiring extremely long and accurate reads to discriminate between amplicon copies and produce a correct reference sequence.

We developed our Single Haplotype Iterative Mapping and Sequencing (SHIMS) approach to cope with the ampliconic sequences of the human Y chromosome [3]. Because paralogous ampliconic repeats are more similar than alleles, we sequenced large-insert clones from a single haplotype, allowing us to confidently identify the rare sequence family variants (SFVs) that distinguish paralogous repeats in highly accurate (less than 1 error per megabase) synthetic long reads [3]. Mapping and sequencing were coupled; newly sequenced clones provide novel SFVs that refine the clone map and serve as markers to select new clones. SHIMS has been instrumental to producing reference sequences of structurally complex sex chromosomes from several species [4–10], as well as the human immunoglobulin gene cluster [11], and other structurally complex regions on human autosomes [12]. SHIMS remains the only sequencing approach that can reliably disentangle ampliconic repeats. Whole genome shotgun (WGS) strategies are constrained by a tradeoff between read length and accuracy among existing sequencing technologies. Sanger or Illumina reads are accurate, but are not long enough to traverse interspersed repeats, much less ampliconic sequence [13]. Single-molecule sequencing technologies like PacBio or nanopore sequencing offer reads long enough to span interspersed repeats and smaller ampliconic sequences, but lack the accuracy to disentangle nearly identical ampliconic repeats [14]. As originally implemented, SHIMS 1.0 required the resources of a fully-staffed genome center to generate Sanger reads, assemble draft sequences, and manually ‘finish’ each clone. We developed SHIMS 2.0 to combine the advantages of a hierarchical clone-based strategy with high-throughput sequencing technologies, allowing a small team to generate sequence, while reducing time and cost by two orders of magnitude, while maintaining high accuracy [15]. However, SHIMS 2.0 still required intensive manual review to resolve internally repetitive clones, and in some cases–particularly short, nearly perfect, tandem repeats–complete resolution remained impossible.

**Fig 1:**
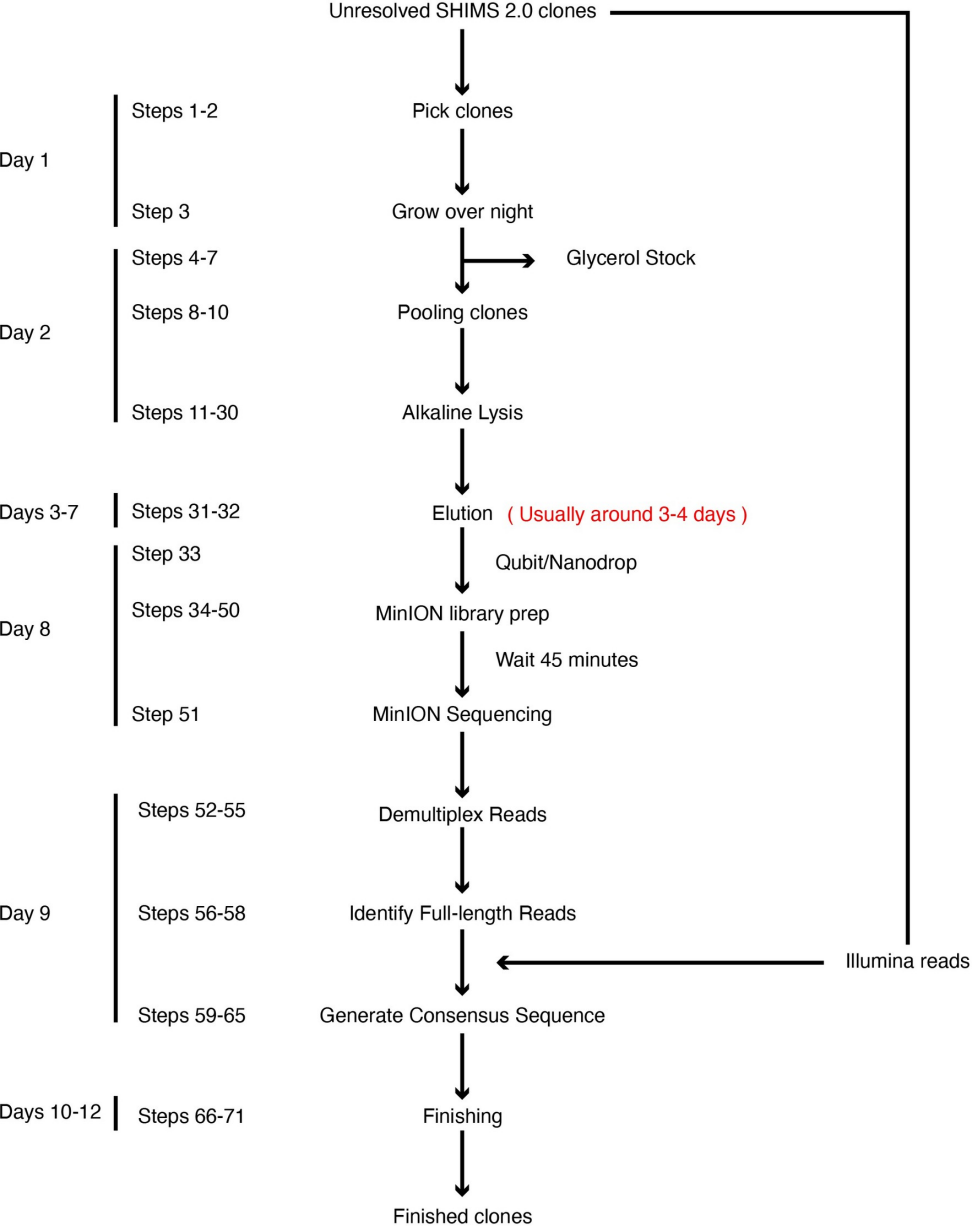
Overview of the SHIMS3.0 protocol. A timeline of a single iteration of the SHIMS 3.0 protocol, showing the major protocol steps, with key quality controls on the right. During a two-week iteration, 24 clones are processed in parallel to rapidly generate finished sequence from structurally complex clones. A single technician can proceed from a list of clones to full-length nanopore libraries in 8 d. After a brief MinION run overnight, a bioinformatics specialist can demultiplex fastq sequences, identify full-length reads, then polish and edit the consensus of these reads to generate finished clone sequence.

Here we describe SHIMS 3.0, an improvement of our SHIMS sequencing strategy that we recently employed to produce reference sequence of structurally complex regions of chimpanzee and rhesus macaque X chromosomes [10]. SHIMS 3.0 uses a combination of nanopore and Illumina sequencing technologies to resolve repetitive structures within individual large-insert clones. We describe a protocol for generating full-length nanopore reads for pools of clones, and combining the structural information from these full-length reads with highly accurate short-read data to automatically produce assemblies of internally repetitive clones (**Fig 1**). This protocol proceeds from clone-picking to finished assemblies in 2 weeks for about $80 (USD) per clone, an improvement of 2 orders of magnitude compared with 24 months and $4000 (USD) under SHIMS 1.0. This protocol makes SHIMS broadly accessible, so that even small teams with no prior experience can produce highly accurate sequence from challenging targets, and opens the way for improved reference sequences of complex regions, including the ampliconic regions of sex chromosomes, across diverse organisms. These reference sequence improvements will have immediate impact, as re-mapping existing short-read datasets to fully resolved ampliconic structures will yield new biological insights.

### Methodology

Large-insert clone libraries derived from a single haplotype are essential to the SHIMS strategy, and are discussed in detail in our description of SHIMS 2.0 [15]. In brief, any library derived from an individual of the heterogametic sex will provide a single haplotype source for sequencing sex chromosomes, albeit at half the coverage of the autosomes. Libraries created from inbred strains can provide a single-haplotype source for autosomes. When inbreeding is not possible, special measures may be necessary to obtain a single-haplotype source of DNA [12]. The ideal library will have greater than 10x coverage of the chromosome of interest to minimize the number of gaps in library coverage. In SHIMS 1.0 and 2.0 it was important to match the average library insert size to the expected amplicon unit size, such that it was rare for two units to be present in the same clone [15]. SHIMS 3.0 uses ultra-long nanopore reads to span entire BAC clones [16]; therefore, we now recommend striving for the largest possible insert size, to minimize library construction, screening, and sequencing costs, as fewer clones will be necessary to achieve the required level of coverage.

All SHIMS strategies begin by selecting an initial tiling path of large-insert clones for sequencing and iterative refinement. Depending on the resources available for each library, it may be possible to identify clones of interest electronically, using fingerprint maps or end sequences; screening high-density filters by hybridization with labeled oligos, or high-dimensional pools for sequence-tagged-site content by PCR. It is most cost-effective to confirm the identity of each clone by generating draft sequence with the SHIMS 2.0 protocol, rather than designing specific assays for each clone [15]. In brief, this highly parallel method involves shearing BAC DNA to generate large (~1 kb) fragments for individually indexed Illumina TruSeq–compatible libraries to sequence and assemble pools of 192 clones in a single week [15]. In our experience, the structure of ampliconic regions is often unclear until a nearly complete tiling path is assembled, as the sequence map gradually unfolds as new variants are identified by sequencing. It is therefore preferable to seed the first iteration with as many clones as possible to identify sequence family variants early, and minimize the total number of iterations.

Draft clone assemblies generated from Sanger or Illumina reads are accurate enough to identify sequence family variants, and identify a minimum tiling path of clones. In previous iterations of SHIMS, each clone in this path would be painstakingly ‘finished’ to produce as correct and contiguous a sequence as possible. Highly skilled technicians would inspect draft assemblies for errors and anomalies, order and orient all draft sequence contigs, close all gaps, and resolve or annotate all sequence ambiguities (e.g. simple sequence repeats). SHIMS 3.0 departs from this approach, instead relying on the ability of nanopore-based sequencing technology to generate full-length reads to scaffold short-read assemblies and eliminate the need for laborious and time-consuming experiments, such as subcloning, PCR reactions, restriction digests, and transposon bombing, that were used to correct draft assemblies in the past.

We have employed SHIMS 3.0 on regions where we have prior knowledge of ampliconic structures that would render sequence assembly from short reads intractable (e.g. conserved palindromes on primate X chromosomes). When sequencing a novel ampliconic target, it is often the case that the underlying genomic structures are known to be too complex for whole genome shotgun sequencing, but the exact repetitive structure is not known. In this case, we recommend sequencing a redundant tiling path of clones by SHIMS 2.0 first, and to deploy SHIMS 3.0 on the subset of clones that fail to assemble automatically from Illumina reads alone. This strategy provides the least expensive means for assembling the full range of ampliconic structures that we have encountered in vertebrate genomes, from tandem arrays of repeats tens of kilobases long to multi-megabase palindromes.

We adapted existing methods for generating ultra-long reads [16] for use with pools of large-insert clones on the Oxford Nanopore Technologies (ONT) MinION platform. Successful generation of full-length reads requires intact DNA of high concentration and purity. We optimized our protocol to avoid unnecessary manipulations that could damage DNA or introduce contamination. We culture 24 clones separately, and then pool all cultures for DNA isolation, library preparation, and sequencing. In contrast to conventional plasmids, BACs and fosmids are present in only a single copy per host cell, and common reagents for increasing the efficiency of DNA precipitation, such as glycogen or SPRI beads, are incompatible with nanopore sequencing. We compensate for this by starting with large volume (~15 ml) BAC and fosmid cultures to ensure that we harvest a sufficient amount of intact DNA. To preserve the integrity of high-molecular-weight (HMW) DNA, we handle it as little as possible, pipetting very slowly, using only wide-bore tips. We allow precipitated DNA to resuspend in water slowly over several days, rather than mixing by vortexing, or pipetting up-and-down. We have had the best results generating libraries from 7.5 to 15 μg of HMW DNA in 15 μl using the transposase-based RAD-004 library preparation kit from ONT. At these concentrations, solutions of HMW DNA will be extremely viscous, and it is difficult to measure the concentration precisely; some trial-and-error may be required to get the correct ratio of transposase to DNA, but we find that 0.5 μl of FRA for 15 μg is generally a good starting point to ensure that most BAC clones are cut only once. It is important to wait 45 minutes between loading the nanopore flow cell and starting the run, to allow time for full-length molecules to diffuse to the pores, otherwise the run will be dominated by shorter molecules. In contrast to our approach for generating Illumina reads, indexing or barcoding individual clones is not necessary, as full-length nanopore reads can be uniquely assigned to clones, even within the same amplicon.

**Fig 2:**
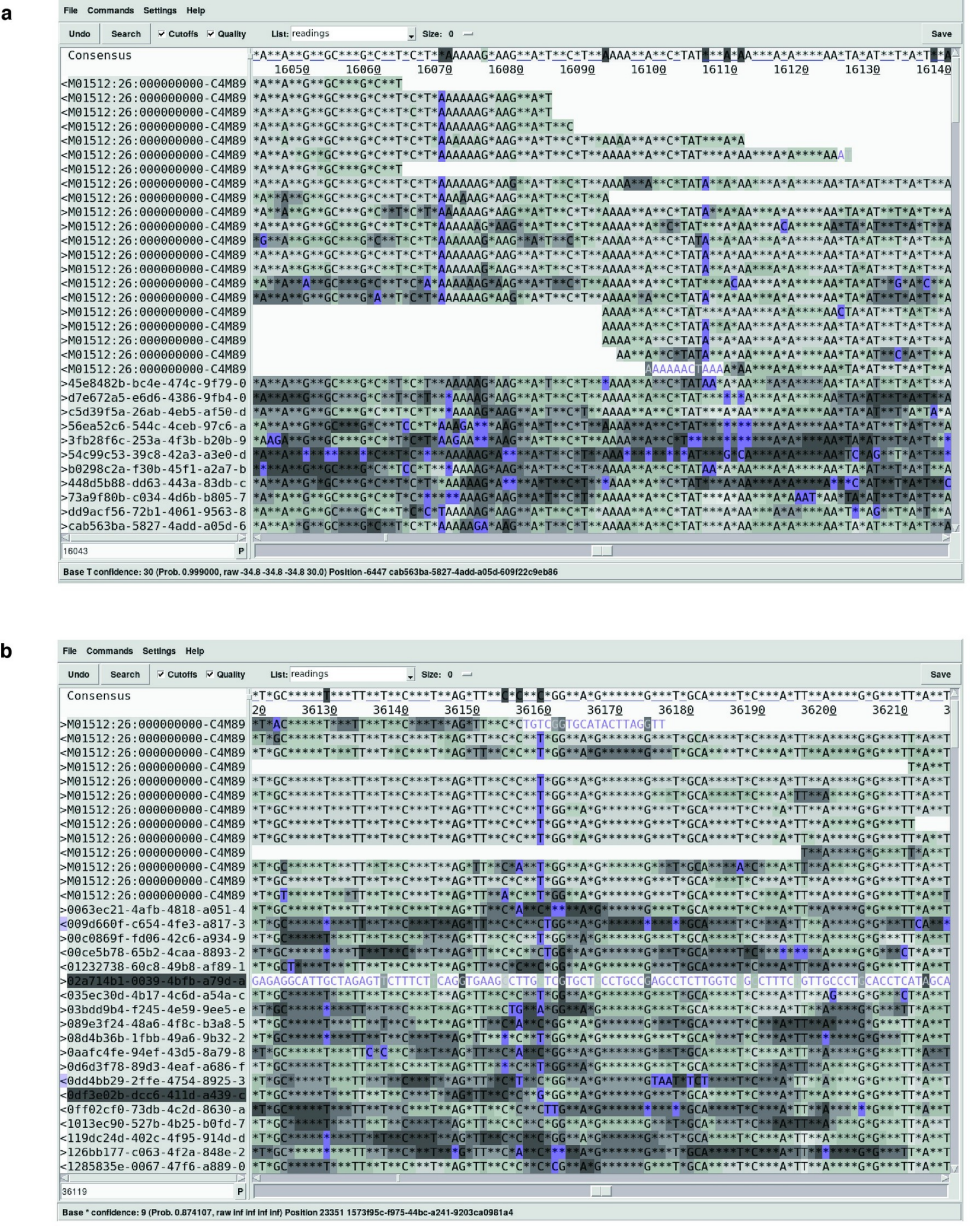
Editing clone assemblies in Gap5. Screenshots from Gap5 with reads sorted by technology (Illumina on top; nanopore on bottom), showing two instances where errors in the consensus can be resolved by correcting to the consensus of the Illumina reads: a) frequent insertion and deletion errors at homopolymer runs, and b) more rare substitution errors.

Full-length nanopore reads transform clone finishing into a purely computational exercise. The tool chain for handling ultra-long nanopore reads is not yet fully mature, but it is developing rapidly. We rely on Minimap2 for alignments involving full-length reads [17]. This includes assigning nanopore reads to clones based on SHIMS 2.0 draft sequences, identifying full-length reads that start and end in vector sequence, and aligning a mix of long and short reads to generate a consensus. We use Racon for polishing the consensus sequence [18], and SAMtools and custom scripts to manipulate read alignments [19]. We use Gap5 [20] and Consed [21] for visualizing discrepant bases and manually editing the consensus (**Fig 2**). While full-length reads guarantee the correct overall sequence structure, a variety of alignment artifacts may occur in clones with highly identical internal repeats. In this case, we find it is best to electronically split the clone sequence into individual repeat units, and correct each unit separately, before merging them together to create a finished clone sequence.

### Performance

Using only Sanger or Illumina reads, the presence of multiple amplicon copies within a single insert causes the clone assembly to collapse. By incorporating full-length nanopore reads, SHIMS 3.0 permits a small team–a technician and bioinformatics specialist–to finish these challenging sequencing targets. Our SHIMS 3.0 protocol can improve 24 SHIMS 2.0 draft sequences to finished quality in 2 weeks at a cost of $80 (USD) per clone. Despite the enormous reduction in staffing, cost, and time, the quality of finished sequence remains extremely high. We observe less than 1 error per megabase in overlaps between clones, on par with previous versions of SHIMS.

### Comparison with other methods

SHIMS produces de novo sequence assemblies with higher accuracy than any other technique, making it possible to produce accurate reference sequence of the most extreme repetitive regions, from ampliconic sequences like the nearly-perfect multi-megabase duplications on the mouse Y chromosome [9], to the thousands of centromeric satellite repeats that form the centromere of the human Y chromosome [22]. This extremely high accuracy is due to the clone-based nature of SHIMS. Each clone represents a single long molecule that can be sequenced repeatedly, with complementary technologies, to generate an assembly that is accurate at the level of overall structure as well as the identity of individual nucleotides. This property makes it possible to identify and repeatedly verify the rare sequence family variants that distinguish ampliconic repeats, and build a high-confidence map from individual clones.

The impressive advances in single-molecule sequencing technologies that enabled SHIMS 3.0 have also increased the capabilities of whole genome shotgun approaches [23]. It is now routine to generate nanopore sequencing runs where half of the bases are in reads longer than 100 kb, so that interspersed repeats and smaller ampliconic structures can be spanned by a single long read. Whole genome shotgun with nanopore reads enabled the complete assembly of the human X chromosome from a single haplotype source, the CHM13hTERT cell line [24]. This effort required deep coverage from nanopore reads as well as from a broad array of complementary sequencing and mapping technologies, combined with manual review of structurally complex regions [24]. Error rates were still orders of magnitude higher than clone based strategies– 1 error in 10 kb in single-copy sequence, and 7 errors per kilobase in sequences present in more than one copy [24]. This elevated error rate in multi-copy sequence is due to the inherent difficulties of uniquely mapping short reads to long paralogous repeats; instead of reconstructing the true sequence, error correction with short reads blurs all paralogs together into an erroneous consensus. A second, orthogonal quality control measure indicates that shotgun sequencing with nanopore reads still lags behind clone-based approaches; 18% of CHM13 BAC sequences from segmental duplications and other difficult-to-assemble regions were missing from the whole genome shotgun assembly of CHM13hTERT [24]. Recently, improvements in shotgun assembly algorithms to take advantage of the length (10–25 kb) and accuracy (>99.9%) of PacBio HIFI reads have achieved even more impressive performance, resolving all but 1% of BAC sequences selected from difficult-to-assemble regions in CHM13 [25]. Nevertheless, segmental duplications contributed disproportionately to breaks in contiguity; about half of all contig ends mapped to known segmental duplications [25]. This suggests that while sequencing technologies continue to improve in read length and accuracy, clone-based approaches will continue to be relevant for generating highly accurate reference sequence, particularly in otherwise inaccessible ampliconic regions.

Relative to previous versions of SHIMS, SHIMS 3.0 greatly reduces the resources required to successfully generate finished sequence. Under SHIMS 1.0, the cost to produce draft sequence averaged about $5000 (USD) per clone, while finishing averaged around $4000 (USD). Weeks of bench experiments and many days of expert review were required to transform each low coverage (5-8x) Sanger draft sequence with frequent gaps into a complete and contiguous assembly. In SHIMS 2.0, we reduced the need for costly finishing activities by opting for much higher coverage (50-80x) in much cheaper Illumina reads. We encounter fewer coverage gaps at this higher depth, and also fewer library gaps because of differences in the library preparation protocol. We rely on sonication to provide random shearing, and amplify library fragments by PCR, as opposed to cloning fragments in *E*. *coli*. Although this deep and relatively even coverage ensured that wet-bench experiments were rarely required for finishing, structurally complex clones still required several days of expert review using an assembly editor like Consed. In the most complex cases, involving many paralogous repeats within a single clone, such as the *TSPY* array or centromeric satellite repeats of the human Y chromosome, it was still impossible to completely resolve the correct structure.

By incorporating full-length nanopore reads from each clone, SHIMS 3.0 now makes it possible to assemble even the most internally repetitive clones. Full-length nanopore reads provide complete certainty about the overall clone structure; there is no doubt about the order and orientation of sequences, and no question about the copy number of complex repeats. This limits finishing activity to the simple matter of resolving the few remaining discrepancies between nanopore and Illumina reads. In most clones, this requires less than an hour of effort for even inexperienced finishers, and results in highly accurate sequences, with less than 1 error per megabase. Highly repetitive clones require more attention, but they can be resolved by a simple divide-and-conquer strategy, where each paralogous repeat is finished separately, with special attention to SFV sites, and then merged to create the full finished sequence. Correctly mapping short reads to repeated sequences becomes more difficult as the number of paralogs increases, increasing the chances that each paralogous repeat unit is blurred toward the consensus. In contrast to WGS strategies, in SHIMS 3.0, this blurring is confined to the boundaries of a single clone, and comparisons with neighboring clones can be used to resolve the position of paralogous SFVs. SHIMS 3.0 dramatically decreases the time, cost, and effort required to obtain finished sequence; using an optimized protocol for preparing HMW DNA in parallel from pools of BAC clones, a small team can finish 24 Illumina draft assemblies in 2 weeks for $80 (USD) per clone.

### Limitations of SHIMS 3.0

SHIMS 3.0 exceeds the capabilities of previous iterations of the SHIMS technique, providing access to the longest, most highly identical ampliconic sequences, as well as arrays of repeated sequences shorter than a single clone. However, SHIMS 3.0 shares two of the same limitations as previous versions of SHIMS and other clone-based approaches. First, the maximum size of BAC inserts limits SHIMS to resolving duplications with <99.999% identity. This limitation will remain until long-read technologies are able to surpass BAC sequencing in both read length and accuracy, or a reliable cloning technology emerges that exceeds the insert size of BACs. Second, SHIMS is limited to sequences that can be cloned into *E*. *coli*. Sequences that are toxic to *E*. *coli* are underrepresented in BAC and fosmid libraries. These library gaps can be resolved by directed efforts that avoid cloning in *E*. *coli*, like sequencing long-range PCR products [4], or using the emerging selective sequencing (“ReadUntil”) capability of nanopore-based sequencers to enrich for reads flanking the gap.

Practitioners of SHIMS 3.0 also face new challenges due to their reliance on nanopore reads spanning 100–300 kb BAC inserts. Bioinformatics tools for aligning, visualizing, and editing reads of this length are not fully mature. SAM and BAM files both encode alignment details in the CIGAR format, however, the BAM format is limited to 65535 operations, which is frequently too few to encode the many transitions between matches, mismatches, insertions and deletions encountered in alignments of ultra-long nanopore reads [26]. Moreover, Consed does not reliably display alignments of reads longer than 1 kb. Our workaround has been to split SAM formatted alignments of nanopore reads into uniquely named sub-alignments every 1000 match operations, and convert the resulting SAM files to BAM format, which is accepted by Consed. This permits full visualization of nanopore reads alongside Illumina reads during finishing.

### Expertise

As with our previous protocol for SHIMS 2.0, we have designed the SHIMS 3.0 protocol to be carried out by a small team. A single technician can process 24 BAC clones from frozen stocks to nanopore sequencing libraries in 5 days with common molecular biology lab equipment. A bioinformatics specialist can set up a pipeline to identify full-length reads for each clone, generate a consensus sequence, automatically correct most errors using alignments with short reads, and manually review the resulting assembly for errors and identify SFVs. It is important to keep abreast of new developments in software for processing nanopore data, as all aspects from base-calling to alignment and error correction are continuously being improved.

## Materials and methods

The protocol described in this peer-reviewed article is published on protocols.io, https://dx.doi.org/10.17504/protocols.io.b34tqqwn and is included for printing as S1 File with this article.

## Expected results

We typically pool 24 clones for a single MinION run, generating about 300,000 reads with a read n50 of 20 kb, and a total of about 1.5 Gb of sequence data. Each clone typically receives 1–5% of the total reads. Occasionally some clones will have no reads; this will become apparent at step 55, and usually indicates that the culture of the clone (Steps 1–3) has failed (**Table 1**).

**Table 1:** Troubleshooting table.

Step	Problem	Possible Reason	Solution
33	Low DNA concentration	Culture undergrowth or overgrowth	Check culture OD600 is between 0.2–0.35
		Incomplete Lysis	Make sure to thoroughly mix the solution until the color is uniform
		Incomplete neutralization	Solution from step 13 should not appear viscous and precipitate should float to the surface
		Incomplete DNA elution	Pre-warm elution buffer to 50°C
34	Concentration varies when checking with NanoDrop or Qubit	DNA is not completely mixed	After adjusting concentration from step 33, leave DNA solution on a heated shaker at the gentlest setting at 50°C until DNA is completely mixed
51	Pores decrease rapidly	Impure DNA sample	Re-check DNA concentration. Extract DNA again if NanoDrop and Qubit results are discordant, 260/280 < 1.7, 260/280 > 2.0, 260/230 < 2.0, or 260/230 > 2.2
		Bubbles introduced during loading	Pipette very slowly and take care not to introduce bubbles during flow cell priming and library loading
55	No reads for one or more clones	Clone culture failed	Regrow and add to the next run, or replace the clone with another
			Regrow the clone for an additional round of sequencing
		Bookkeeping error; some common bookkeeping errors result from transposing digits, rotating a plate by 180°, or contamination from a clone in an adjacent well	Resolve bookkeeping error, and rerun a new clone or replace with another clone
58	Low fraction of long reads	FRA treatment time too long	Promptly heat-inactivate FRA at 35 seconds
			Adjust the FRA incubation time below 35 seconds
		Shearing during library prep	Use wide-bore tips for all mixing and loading steps
71	Clone sequence is shorter than expected or missing known sequence	Deletion during culture	Regrow the clone from the original culture or another library copy, and replace with the alternate clone
		Sequence toxic to *E*. *coli*	Close the gap by long-range PCR or region-specific extraction

Expect to obtain 3–10 full-length reads per clone. Because of the high rate of insertions and deletions in individual nanopore reads, full-length reads may differ in length by 10 kb or more. Occasionally, a clone will have no reads that start and end in vector sequence, but the clone length will be apparent from a peak in the tail of the distribution of read lengths. It may still be possible to reconstruct a full-length consensus sequence by rotating one of these putative full-length reads to place the vector sequence at the beginning. However, we do not recommend this procedure for internally repetitive clones, particularly tandem arrays. Instead, sequence the clone again, and use these ambiguous reads to help polish the consensus.

## Supporting information

S1 File Step-by-step protocol, also available on protocols.io. (PDF)
