# Supplementary material for: SHIMS 3.0: Highly efficient single-haplotype iterative mapping and sequencing using ultra-long nanopore reads
Source: PLoS One. 2022 Jun 14;17(6):e0269692. doi: 10.1371/journal.pone.0269692 (PMC9197060; doi:10.1371/journal.pone.0269692)
Supplement: S1 File — (PDF) [file pone.0269692.s001.pdf]

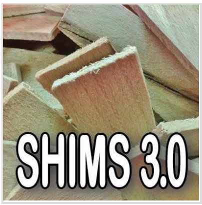

2

SHIMS 3.0: Highly efficient single-haplotype iterative mapping and sequencing using ultra-long nanopore reads V.2

Daniel W Bellott[1], Ting-Jan Cho[1], Emily K Jackson[2], Helen Skaletsky[1,3], Jennifer F. Hughes[1], David C Page[1,2,3]

[1]Whitehead Institute, Cambridge, Massachusetts, USA;

[2]Department of Biology, Massachusetts Institute of Technology, Cambridge, Massachusetts, USA;

[3]Howard Hughes Medical Institute, Whitehead Institute, Cambridge, Massachusetts, USA

dx.doi.org/10.17504/protocols.io.b34tqqwn

Daniel Bellott

Acknowledgements
This work was supported by the Howard Hughes Medical Institute, and generous gifts from Brit and Alexander d'Arbeloff and Arthur W. and Carol Tobin Brill

Author Contributions
D.W.B., H.S., J.F.H., and D.C.P. designed the study. D.W.B., T.-J.C., and E.K.J. developed the experimental methods. D.W.B. wrote the scripts for computational analysis. D.W.B., T.-J.C., and D.C.P. wrote the manuscript.

Competing Interests
The authors declare no competing interests.

Code Availability
We have automated Steps 56-65 with a custom Perl script, available at:
https://github.com/dwbellott/shims3_assembly_pipeline/

The reference sequence of structurally complex regions can only be obtained through a highly accurate clone-based approach that we call Single-Haplotype Iterative Mapping and Sequencing (SHIMS). In recent years, improvements to SHIMS have reduced the cost and time required by two orders of magnitude, but internally repetitive clones still require extensive manual effort to transform draft assemblies into reference-quality finished sequences. Here we describe SHIMS 3.0, using ultra-long nanopore reads to augment the Illumina data from SHIMS 2.0 assemblies and resolve internally repetitive structures. This greatly minimizes the need for manual finishing of Illumina-based draft assemblies, allowing a small team with no prior finishing experience to sequence challenging targets with high accuracy. This protocol proceeds from clone-picking to finished assemblies in 2 weeks for about 80 dollars per clone. We recently used this protocol to produce reference sequence of structurally complex palindromes on chimpanzee and rhesus macaque X chromosomes. Our protocol provides access to structurally complex regions that would otherwise be inaccessible from whole-genome shotgun data or require an impractical amount of manual effort to generate an accurate assembly.

Figure_1.pdf Figure_2.pdf

DOI

dx.doi.org/10.17504/protocols.io.b34tqqwn

Daniel W Bellott, Ting-Jan Cho, Emily K Jackson, Helen Skaletsky, Jennifer F. Hughes, David C Page . SHIMS 3.0: Highly efficient single-haplotype iterative mapping and sequencing using ultra-long nanopore reads. **protocols.io** https://dx.doi.org/10.17504/protocols.io.b34tqqwn
Daniel Bellott

BAC, Nanopore, MinION, consed, gap5, sequencing, assembly, SHIMS , genome

protocol ,

Jan 20, 2022

Jan 20, 2022

57203

TIMING

Steps 1-3, pick clones and grow cultures: **18:00:00**

Steps 4-7, glycerol stock plate: **00:30:00**

Steps 8-10, pooling clones: **01:00:00** - **02:00:00**

Steps 11-33, alkaline lysis: **01:00:00** - **02:00:00**

Steps 34-42, MinION library prep: **00:30:00**

Steps 43-51, MinION library loading: **00:30:00**

Steps 52-55, demultiplex reads: **00:30:00**

Steps 56-58, Identify full-length reads: **00:30:00**

Steps 59-65, generate consensus sequence: **00:30:00**

Steps 66-71, finishing: **00:00:00** - **08:00:00**

A	B	C	D	E
Step	Problem	Possible Reason	Solution	
33	Low DNA concentration	Culture undergrowth or overgrowth	Check culture OD600 is between 0.2-0.35	

		Incomplete Lysis	Make sure to thoroughly mix the solution until the color is uniform	
		Incomplete neutralization	Solution from step 13 should not appear viscous and precipitate should float to the surface	
		Incomplete DNA elution	Pre-warm elution buffer to 50°C	
34	Concentration varies when checking with NanoDrop or Qubit	DNA is not completely mixed	After adjusting concentration from step 33, leave DNA solution on a heated shaker<br>at the gentlest setting at 50°C until DNA is completely mixed	
51	Pores decrease rapidly	Impure DNA sample	Re-check DNA concentration. Extract DNA again if NanoDrop and Qubit results are discordant, 260/280 < 1.7, 260/280 > 2.0, 260/230 < 2.0, or 260/230 > 2.2	
		Bubbles introduced during loading	Pipette very slowly and take care not to introduce bubbles during flow cell priming and library loading	
55	No reads for one or more clones	Clone culture failed	Regrow and add to the next run, or replace the clone with another	
			Regrow the clone for an additional round of sequencing	
		Bookkeeping error; some common bookkeeping errors result from transposing digits, rotating a plate by 180°, or contamination from a clone in an adjacent well	Resolve bookkeeping error, and rerun a new clone or replace with another clone	
58	Low fraction of long reads	FRA treatment time too long	Promptly heat-inactivate FRA at 35 seconds	
			Adjust the FRA incubation time below 35 seconds	
		Shearing during library prep	Use wide-bore tips for all mixing and loading steps	

71	Clone sequence is shorter than expected or missing known sequence	Deletion during culture	Regrow the clone from the original culture or another library copy, and replace with the alternate clone	
		Sequence toxic to E. coli	Close the gap by long-range PCR or region-specific extraction	

Table 1 | Troubleshooting Table

Anticipated Results

We typically pool 24 clones for a single MinION run, generating about 300,000 reads with a read n50 of 20 kb, and a total of about 1.5 Gb of sequence data. Each clone typically receives 1-5% of the total reads. Occasionally some clones will have no reads; this usually indicates that the culture of the clone (Steps 1-3) has failed (see troubleshooting information for Step 55).

Expect to obtain 3-10 full-length reads per clone. Because of the high rate of insertions and deletions in individual nanopore reads, full-length reads may differ in length by 10 kb or more. Occasionally, a clone will have no reads that start and end in vector sequence, but the clone length will be apparent from a peak in the tail of the distribution of read lengths. It may still be possible to reconstruct a full-length consensus sequence by rotating one of these putative full-length reads to place the vector sequence at the beginning. However, we do not recommend this procedure for internally repetitive clones, particularly tandem arrays. Instead, sequence the clone again, and use these ambiguous reads to help polish the consensus.

MATERIALS

REAGENTS

Ethyl Alcohol 200 Proof **PHARMCO-AAPER™ Catalog #11000200** (Ethanol)

!CAUTION Ethyl Alcohol is flammable; keep away from flame when handling it.

Tris Base **American Bio Catalog #AB020000-05000**

Hydrochloric Acid **VWR International Catalog #BDH3026-500MLP** (HCl)

!CAUTION Hydrochloric Acid is corrosive. Wear gloves and eye protection when handling it.

Polyethylene glycol 8000 **Sigma Aldrich Catalog #P2139-2KG** (PEG-8000)

Sodium Chloride **American Bio Catalog #AB01915-10000** (NaCl)

Bacto-tryptone **BD Biosciences Catalog #211705**

Yeast Extract **BD Biosciences Catalog #211929**

Chloramphenicol **Sigma Aldrich Catalog #C0378-5G**

!CAUTION Chloramphenicol powder is hazardous. Handle this reagent in ventilated fume hood with gloves and eye protection.

Glycerol **Emd Millipore Catalog #356350-1000ML**

ZymoPURE II Plasmid Maxiprep Kit **Zymo Research Catalog #D4203**

Rapid Sequencing Kit **Oxford Nanopore Technologies Catalog #SQK-RAD004**

EQUIPMENT

EZ-Vac Vacuum Manifold
Vacuum Manifold
Zymo Research S7000

MinIon Mk1B
Sequencer

Oxford Nanopore Technologies **MIN-101B**

NanoDrop 1000 Spectrophotometer
Spectrophotometer

Nanodrop **ND 1000**

Centrifuge 5810R
Benchtop Centrifuge

Eppendorf **00267023**

Microsentrifuge 5425
Microcentrifuge

Eppendorf **5405000107**

Vortex-Genie 2
Vortex Mixer

Scientific Industries **SI-0236**

Portable Pipet-Aid® XP2 Pipette Controller
Pipette Controller

Drummond **4-000-501**

Eppendorf ThermoMixer® F
Mixer

Eppendorf **5380000028**

Avanti J-E BioSafe Centrifuge System, 50 Hz, 230 V
High-speed Centrifuge

Beckman Coulter **A20698**

Fisherbrand Low-Retention Microcentrifuge Tubes
Microcentrifuge Tubes

Fisherbrand **02-681-320**

ART™ Wide Bore Filtered Pipette Tips
Filtered Pipette Tips

Thermo Scientific **2069GPK**

Falcon 50mL Conical Centrifuge Tubes
Centrifuge Tubes

Falcon 14-959-49A

Thermo Scientific™ Nalgene™ PPCO Centrifuge Bottles with Sealing Closure
Centrifuge Bottles

Thermo Scientific 3141-0250

Corning® 96-well EIA/RIA Clear Round Bottom Polystyrene Not Treated Microplate, 25 per Bag, without Lid, Nonsterile
Microplate

Corning 3797

Nunc™ 96-Well Polypropylene DeepWell™ Storage Plates
Microplate

Thermo Scientific 278743

AirPore Tape Sheets
Microplate Seal

Qiagen 19571

Parafilm M film
Laboratory Wrapping Film
Bemis 13-374-10

Microseal® 'F' PCR Plate Seal, foil, pierceable
Plate Seal
Bio-Rad MSF1001

SOFTWARE

minimap2 2.17
Ubuntu 20.04.3 LTS
source by Heng Li

racon 1.4.17
Ubuntu 20.04.3 LTS
source by Ivan Sovic

samtools 1.11
Ubuntu 20.04.3 LTS
source by Heng Li

tg_index 1.2.13-r
Ubuntu 20.04.3 LTS
source by James K. Bonfield, Andrew Whitwham

gap5 1.2.14-r
Ubuntu 20.04.3 LTS
source by James K. Bonfield, Andrew Whitwham

Optional:

consed 29.0
Ubuntu 20.04.3 LTS
by David Gordon

REAGENT SETUP

70% (vol/vol) Ethanol

Mix **30 mL** of [M]**100 % (v/v)** ethanol with **70 mL** of of distilled, deionized water (ddH$_2$O).

CRITICAL 70% (vol/vol) ethanol should be prepared on the day of the experiment.

1M Tris-Cl, pH 8.5

Dissolve **121 g** of Tris base in **800 mL** of ddH$_2$O. Adjust pH to pH **8.5** with concentrated HCl, then adjust volume with ddH$_2$O to **1 L** . 1M Tris-Cl can be prepared in advance and stored at **Room temperature** (**22 °C**) for up to 1 year.

10 mM Tris-Cl, pH 8.5

Mix **0.5 mL** of [M]**1 Molarity (M)** Tris-Cl with **49.5 mL** of ddH$_2$O. This solution can be prepared in advance and stored at **Room temperature** for up to 1 year.

PEG buffer (18% PEG(wt/vol)/1M NaCl Solution)

Add **135 g** of PEG-8000 powder into **1 L** bottle. Add **150 mL** of [M]**5 Molarity (M)** NaCl, **7.5 mL** of Tris-HCl, **1.5 mL** of [M]**0.5 Molarity (M)** EDTA and **450 mL** of ddH$_2$O to make PEG buffer. Store at **Room temperature** for up to 1 year.

2X LB

Add **20 g** of bacto-tryptone, **10 g** of yeast extract, and **20 g** of NaCl to ddH2O, and adjust the volume to **1 L** . Mix well with a magnetic stirrer. After mixing, distribute **500 mL** aliquots into **1 L** bottles. Cap loosely, prewarm to **50 °C** , and autoclave for

00:20:00 on liquid cycle. Store at Room temperature for up to 1 year.

Chloramphenicol

Dissolve 0.34 g of chloramphenicol into 10 mL of [M] 100 % (v/v) ethanol.

Chloramphenicol stock can be stored at -20 °C for up to 1 year.

80% (vol/vol) Glycerol solution

Add 400 mL of glycerol to a graduated cylinder; adjust the volume to 500 mL with ddH2O. Seal the cylinder with Parafilm M film, and mix by inversion. Transfer to a bottle, and autoclave for 00:20:00 in liquid cycle. This solution can be prepared in advance and stored at Room temperature for up to 1 year

!CAUTION Ethyl Alcohol is flammable; keep away from flame when handling it.
!CAUTION Hydrochloric Acid is corrosive. Wear gloves and eye protection when handling it.
!CAUTION Chloramphenicol powder is hazardous. Handle this reagent in ventilated fume hood with gloves and eye protection.

:

Acknowledgements

This work was supported by the Howard Hughes Medical Institute, and generous gifts from Brit and Alexander d'Arbeloff and Arthur W. and Carol Tobin Brill

Author Contributions

D.W.B., H.S., J.F.H., and D.C.P. designed the study. D.W.B., T.-J.C., and E.K.J. developed the experimental methods. D.W.B. wrote the scripts for computational analysis. D.W.B., T.-J.C., and D.C.P. wrote the manuscript.

Competing Interests

The authors declare no competing interests.

Code Availability

We have automated Steps 56-65 with a custom Perl script, available at:
https://github.com/dwbellott/shims3_assembly_pipeline/

Prepare stock solutions described in Materials:
- **1M Tris-Cl, pH 8.5**
- **10 mM Tris-Cl, pH 8.5**
- **PEG buffer (18% PEG(wt/vol)/1M NaCl Solution)**
- **2X LB**
- **Chloramphenicol**

- **80% (vol/vol) Glycerol solution**

Figure 1 provides an overview of the protocol.
Troubleshooting advice can be found in **Table 1**.

Pick Clones and Grow Cultures 18h

1 ⚠

Fill each well of a Nunc 96 DeepWell plate with **1.9 mL** of 2X LB containing [M]**34 µg/ml** chloramphenicol.

CRITICAL STEP
Rich media (2X LB) is appropriate for single-copy plasmids like BACs or fosmids, which use chloramphenicol resistance as a selectable marker.

2 ⚠

Use a clean pipette tip to scrape the surface of a frozen glycerol stock and drop the tip directly into the DeepWell plate to inoculate a well. Inoculate each sample 8 times for a total of **15.2 mL** per sample (8 x **1.9 mL**). 24 samples in total for each library prep.

CRITICAL STEP
Start with a glycerol stock of a clone verified to be correct by PCR for a known sequence-tagged site or previous sequencing experiments (e.g. cultures created during Step 7 of the SHIMS 2.0 protocol).

3 ⚠

Seal plates with AirPore Tape Sheets and incubate on a shaker at **220 rpm, 37°C, 17:00:00** .

CRITICAL STEP
Overgrowth of cultures (cell density > 3 x 10^9 cells per ml) will decrease yield of BAC DNA.

Glycerol Stock Plate 30m

4 Dispense **150 µl** of 80% (vol/vol) glycerol solution into two rows of a Costar Assay Plate.

5 Transfer **150 µl** of each sample culture from Step 3 to a corresponding well of the assay plate and mix by pipetting up and down 20 times.

6 Seal the glycerol stock plate with aluminum adhesive foil.

7 Store the glycerol stock plate at **-80 °C**

Pooling Clones 2h

8 Pour overnight cultures from Step 3 into a large beaker to combine pool.

9 Divide pooled culture into two 250 mL Nalgene bottles and spin down culture at **6000 x g, 4°C, 00:30:00** 30m

10

Remove media by pouring into a waste-collecting container. Be careful not to disturb the pellets.

PAUSE POINT
Store at **-20 °C** for up to a week.

Alkaline Lysis 2h

11 Add **7 mL** of ZymoPURE P1 (Red) to each bacterial cell pellet and resuspend completely by pipetting. Combine into one bottle when cells are completely resuspended.

12

Add 14 mL of ZymoPURE P2 (Green) and immediately mix by gently inverting the tube 6 times.

CRITICAL STEP
Do not vortex! Let sit at Room temperature for 00:03:00 . Cells are completely lysed when the solution appears clear, purple, and viscous.

13 ⚠

Add 14 mL of ZymoPURE P3 (Yellow) and mix gently but thoroughly by inversion.

CRITICAL STEP
Do not vortex! The sample will turn yellow when the neutralization is complete, and a yellowish precipitate will form.

14 Ensure the plug is attached to the Luer Lock at the bottom of the ZymoPURE Syringe Filter. Place the syringe filter upright in a tube rack and load the lysate into the ZymoPURE Syringe Filter and wait 00:08:00 for the precipitate to float to the top. 8m

15 Remove the Luer Lock plug from the bottom of the syringe and place it into a clean 50 mL conical tube. Place the plunger in the syringe and push the solution through the ZymoPURE Syringe Filter in one continuous motion until approximately 33 mL - 35 mL of cleared lysate is recovered. Save the cleared lysate!

16 Add 14 mL ZymoPURE Binding Buffer to the cleared lysate from step 5 and mix thoroughly by inverting the capped tube 10 times.

17 Ensure the connections of the Zymo-Spin V-P Column Assembly are finger-tight and place onto a vacuum manifold.

18 With the vacuum off, add the entire mixture from step 16 into the Zymo-Spin V-P Column Assembly, and then turn on the vacuum until all the liquid has passed completely through the column.

19 Remove and discard the 50 mL reservoir from the top of the Zymo-Spin V-P Column Assembly.

20 With the vacuum off, add **5 mL** of ZymoPURE Wash 1 to the 15 mL Conical Reservoir in the Zymo-Spin V-P Column Assembly. Turn on the vacuum until all the liquid has passed completely through the column.

21 With the vacuum off, add **5 mL** of ZymoPURE Wash 2 to the 15 mL Conical Reservoir. Turn on the vacuum until all the liquid has passed completely through the column. Repeat this wash step.

22 Remove and discard the 15 ml Conical Reservoir and place the Zymo-Spin V-P Column in a Collection Tube. Centrifuge at **10000 x g, 00:01:00** , in a microcentrifuge, to remove any residual wash buffer. 1m

23 Pre-warm **450 µl** of 10 mM Tris-Cl to 50 °C. Transfer the column into a clean 1.5ml microcentrifuge tube and add the **450 µl** of 10 mM Tris-Cl directly to the column matrix. Wait **00:10:00** , and then centrifuge at **10000 x g, 00:01:00** in a microcentrifuge. 11m

24 Add **450 µl** of PEG buffer to the tube containing sample. Mix by flicking and rotating the 1.5 ml microcentrifuge tube.

25 Centrifuge at **10000 x g, 4°C, 00:30:00** , in a microcentrifuge. 30m

26 Remove supernatant from the tube without disturbing the pellet.

27 Add **1 mL** of 70% EtOH and spin **10000 x g, 4°C, 00:10:00** , in a microcentrifuge. 10m

28 Repeat step 26 and 27.

29 Remove supernatant and any left over 70% EtOH from microcentrifuge tube.

30 ⚠ 10m

Air dry for 00:10:00 or until no visible liquid is left in the tube.

CRITICAL STEP
Do not overdry the pellet

31 Dissolve DNA pellet in 18 µl 10 mM Tris-Cl.

32 Store DNA at 4 °C for several days until pellet completely dissolves into solution.

33 Check DNA concentration and quality with Qubit or NanoDrop. Aim for concentration ≥ [M]1 µg/µl, an A260/280 ratio ~ 1.8, and an A260/230 ratio between 2.0 and 2.2.

TROUBLESHOOTING
Troubleshooting advice can be found in **Table 1**.

MinION Library prep 30m

34 Adjust sample concentration from step 33 to [M]1 µg/µl with 10 mM Tris-Cl.

TROUBLESHOOTING
Troubleshooting advice can be found in **Table 1**.

35 ⚠

Using a wide bore pipette tip, slowly aspirate 15 µl into a low retention microcentrifuge

tube.

CRITICAL STEP
Pipetting can shear fragile high-molecular-weight DNA. Pipette slowly using wide bore pipette tips.

36 ⚠

In a separate microcentrifuge tube, add **0.5 µl** FRA to **4.5 µl** 10 mM Tris-Cl. Flick the tube to mix well.

CRITICAL STEP
The FRA solution is included in the Rapid Sequencing Kit.

37 Add the diluted FRA solution from step 36 into sample tube from step 35.

38 ⚠

Gently flick the tubes a few times to mix.

CRITICAL STEP
Vortexing can shear fragile high-molecular-weight DNA. Flick solutions to mix.

39 Incubate sample on **30 °C** heat block for **00:00:35** , then move the tube to **80 °C** heat block. Incubate for **00:02:00** at **80 °C** . 2m 35s

40 Remove the tube from heat block and incubate **On ice** for **00:01:00** , then move the tube off the ice. Equilibrate to **Room temperature** , about **00:01:00** . 2m

41 ⚠

While the sample is equilibrating to **Room temperature** , add **4.5 µl** 10 mM Tris-Cl to **0.5 µl** of RAP. Flick to mix well.

CRITICAL STEP
The RAP solution is included in the Rapid Sequencing Kit.

42 ⚠

Add RAP dilution from step 41 into sample tube. Slowly flick the tube a few times to mix. Keep the sample at **Room temperature** before loading.

CRITICAL STEP
Vortexing can shear fragile high-molecular-weight DNA. Flick solutions to mix.

MinION Library loading 30m

43 ⚠

Add **30 µl** of FLT to one tube of FLB, to make flush buffer, according to the Rapid Sequencing Kit instructions. Vortex the solution to mix , then centrifuge briefly.

CRITICAL STEP
The FLT and FLB solutions are included in the Rapid Sequencing Kit.

44 Perform QC on a new MinION flow cell to check available pores and ensure that a sufficient number of pores are present. If there are fewer than 800 available pores, place the flow cell in storage and use a new MinION flow cell.

45 Use a P1000 pipette to remove about **20 µl** - **30 µl** of storage buffer from priming pore. Load **800 µl** flush buffer via the pore slowly. Wait **00:05:00** . 5m

46 Lift SpotON cover and load **200 µl** flush buffer slowly. Try to dispense at a speed where each bead of liquid is siphoned into the SpotON port as soon as it is visible.

47 ⚠

Add 34 µl SQB and 15 µl ddH2O to the sample tube from step 42.

CRITICAL STEP
The SQB solution is included in the Rapid Sequencing Kit.

48 ⚠

Flick the tube gently to mix, then centrifuge briefly to collect library to the bottom of the tube.

CRITICAL STEP
Vortexing can shear fragile high-molecular-weight DNA. Flick solutions to mix.

49 ⚠

Slowly aspirate 75 µl of library with a wide bore tip. Very slowly, load the library into SpotON pore drop by drop.

CRITICAL STEP
Pipetting can shear fragile high-molecular-weight DNA. Pipette slowly using wide bore pipette tips.

50 Close both priming pores and put the SpotON cover back onto the pore.

51 ⚠ 45m

After loading the library, leave the flow cell on bench for 00:45:00 before starting the run.

CRITICAL STEP

Wait at least 00:45:00 between loading the flow cell and starting the run. This allows time for full-length molecules to diffuse to the pores. Starting the run earlier will favor the sequencing of shorter molecules.

TROUBLESHOOTING

Troubleshooting advice can be found in **Table 1**.

Demultiplex Reads 30m

52 ⚠

Prepare file of draft clone sequences in fasta format:

```
draft_clones.fa
```

CRITICAL STEP

When concatenating draft sequence assemblies, ensure that each sequence has a unique name.

53 Prepare file of vector sequence in fasta format:

```
vector.fa
```

54 Download fastq formatted reads from the device running MinION control software:

```
nanopore.fq
```

55 Align nanopore reads to file of draft sequences to assign nanopore reads to clones by best match. Then, for each clone, “clone_name”, gather a list of matching read names, then use the file of matching read names to gather the matching reads in fastq format:

TROUBLESHOOTING

Troubleshooting advice can be found in **Table 1**.

```
minimap2 -x map-ont draft_clones.fa nanopore.fq | sort -r -n -k 10
| awk '!seen[$1]++' > best_clone_match.paf

grep clone_name best_clone_match.paf | cut -f 1 >clone_name.txt

grep -A 3 -f clone_name.txt nanopore.fq | grep -v "^--$" >
clone_name.nanopore.fq
```

Identify Full-length Reads 30m

56 ⚠

CRITICAL
We have automated Steps **56-65** with a custom Perl script (available at https://github.com/dwbellott/shims3_assembly_pipeline/), but the workflow is described below to allow for direct use of the individual software tools or substitution of alternative tools.

For each clone, align nanopore reads to file of vector sequence:

```
minimap2 -x map-ont vector.fa clone_name.nanopore.fq –o
clone_name.vector.paf
```

57 Search for reads that begin and end with high-quality matches to vector sequence on the same strand – these are full-length reads.

```
cut -f 1,5,6 clone_name.vector.paf | sort | uniq -c | sed ‘s/^//’ |
grep “^2” | cut -f 2 -d ’ ’ >clone_name.2x.txt

awk ‘$2 - $3 < $7 && $12 == 60’ clone_name.vector.paf | cut -f
1,5,6 | grep -f clone_name.2x.txt >clone_name.2x.right.txt

awk ‘$4 < $7 && $12 == 60’ clone_name.vector.paf | cut -f 1,5,6 |
grep -f clone_name.2x.right.txt | cut -f 1 | sort | uniq
>clone_name.fl.txt
```

58 For each clone, generate a fastq file of full-length reads, as well as a fasta file of the longest full-length read to use as a scaffold for final assembly.

[illegible]

TROUBLESHOOTING
Troubleshooting advice can be found in **Table 1**.

```
grep -A 3 -f clone_name.fl.txt clone_name.nanopore.fq | grep -v "^-
-$" > clone_name.fl.fq

grep -A 1 `head -n 1 clone_name.fl.txt` nanopore.fq | sed
's/^\@.*/\>clone_name/' >clone_name.longest.fl.fa
```

Generate Consensus Sequence 30m

59 Polish the longest read twice, using the other full-length nanopore reads:

```
minimap2 -x map-ont clone_name.longest.fl.fa clone_name.fl.fq
>clone_name.longest.fl.paf

racon clone_name.fl.fq clone_name.longest.fl.paf
clone_name.longest.fl.fa >clone_name.longest.fl.racon.fa

minimap2 -x map-ont clone_name.longest.fl.racon.1.fa
clone_name.fl.fq >clone_name.longest.fl.racon.paf

racon clone_name.fl.fq clone_name.longest.fl.racon.1.paf
clone_name.longest.fl.racon.1.fa >clone_name.fl.consensus.fa
```

60 ⚠

Gather up Illumina, nanopore, and (if available) PacBio reads for each clone.

CRITICAL STEP
Ensure that each member of an Illumina read pair has a unique identifier before combining them with unpaired reads.

```
cat clone_name.illumina.forward.fq | awk '1==NR%4{$1=$1"/1"} 1' >>
clone_name.illumina.forward.uq.fq

cat clone_name.illumina.reverse.fq | awk '1==NR%4{$1=$1"/2"} 1' >>
clone_name.illumina.reverse.uq.fq

cat clone_name.illumina.forward.uq.fq
clone_name.illumina.reverse.uq.fq clone_name.illumina.single.fq
clone_name.nanopore.fq clone_name.pacbio.fq >>
clone_name.allreads.fq
```

61 ⚠

Polish the nanopore consensus sequence, using both long and short reads.

CRITICAL STEP
Our SHIMS2.0 protocol identifies paired-end Illumina reads that overlap and generates single consensus sequences using FLASH (http://ccb.jhu.edu/software/FLASH/) . We use the `asm20` alignment preset in minimap2 to align these unpaired sequences. Users with true single-ended Illumina data may wish to use the `sr` preset instead.

```
minimap2 -x asm20 clone_name.nanopore.consensus.fa
clone_name.illumina.single.fq >> clone_name.polish.1.paf

minimap2 -x sr clone_name.nanopore.consensus.fa
clone_name.illumina.forward.uq.fq clone_name.illumina.reverse.uq.fq
>> clone_name.polish.1.paf

minimap2 -x map-ont clone_name.nanopore.consensus.fa
clone_name.nanopore.fq >> clone_name.polish.1.paf

minimap2 -x map-pb clone_name.nanopore.consensus.fa
clone_name.pacbio.fq >> clone_name.polish.1.paf

racon clone_name.allreads.fq clone_name.polish.1.paf
clone_name.nanopore.consensus.fa >clone_name.polish.1.fa
```

62 Repeat Step 61 four more times, for a total of 5 rounds of polishing

63 ⚠

Align reads one last time to generate SAM format alignments suitable for assembly editors.

CRITICAL STEP
The BAM file format cannot accommodate CIGAR strings with greater than 65535 operations. Alignments involving nanopore reads spanning the full length of a BAC clone will exceed this limit. We strongly recommend storing alignments in SAM or CRAM format to avoid the loss of detailed alignment information.

```
minimap2 -x asm20 -a -L --sam-hit-only -R
'@RG\tID:S\tSM:S\tPL:ILLUMINA' clone_name.polish.5.fa
clone_name.illumina.single.fq | samtools sort -O SAM -
>clone_name.single.sorted.sam

minimap2 -x sr -a -L --sam-hit-only -R
'@RG\tID:FR\tSM:FR\tPL:ILLUMINA' clone_name.polish.5.fa
clone_name.illumina.forward.fq clone_name.illumina.reverse.fq |
samtools sort -O SAM - >clone_name.paired.sorted.sam

minimap2 -x map-pb -a -L --sam-hit-only -R
'@RG\tID:P\tSM:P\tPL:PACBIO' clone_name.polish.5.fa
clone_name.pacbio.fq | samtools sort -O SAM -
>clone_name.pacbio.sorted.sam

minimap2 -x map-ont -a -L --sam-hit-only -R
'@RG\tID:N\tSM:N\tPL:PACBIO' clone_name.polish.5.fa
clone_name.nanopore.fq | samtools sort -O SAM -
>clone_name.nanopore.sorted.sam
```

64 Combine alignments

```
samtools merge -f clone_name.allreads.sorted.sam
clone_name.single.sorted.sam clone_name.paired.sorted.sam
clone_name.pacbio.sorted.sam clone_name.nanopore.sorted.sam
```

65 ⚠

Generate database for Gap5

CRITICAL STEP
We now recommend Gap5 over Consed, because Gap5 natively supports loading data directly from SAM files and displaying full-length nanopore reads. It is possible to split SAM alignments of full-length nanopore reads into smaller fragments that can be encoded in a BAM file and displayed by Consed without loss of information. For those who wish to use Consed, we implement this work-around in a custom Perl script (available at https://github.com/dwbellott/shims3_assembly_pipeline/).

```
tg_index -o clone_name.g5d -p -9 -s clone_name.allreads.sorted.sam
```

Finishing 1h

66 Open the assembly in Gap5:

```
gap5 clone_name.g5d
```

67 Select 'Edit Contig' from the 'Edit' menu.

68 ⚠

Resolve discrepancies between Illumina reads and full-length nanopore reads (Fig. 2).

CRITICAL STEP
In Gap5, it is not possible to directly edit the consensus sequence. Instead, indicate which readings are authoritative by marking bases as high quality with the ']' key, and the consensus will automatically update.

CRITICAL STEP
We usually resolve the consensus in favor of the Illumina reads. The vast majority of discrepancies between these technologies occur at homopolymer repeats, where nanopore reads are especially prone to insertion and deletion errors (**Fig. 2a**). More rarely, we encounter systematic errors in nanopore base calling that generate a consensus that is not supported by any Illumina read.

CRITICAL STEP
We resolve disagreements among Illumina reads in favor of the consensus of full-length nanopore reads. In clones that contain duplicated sequences, short Illumina reads can be mapped to the wrong repeat unit, but full-length nanopore reads are not subject to this artifact, and will usually have the correct base at each SFV.

69 ⚠

Resolve SSRs by realigning reads around the SSR region. Select reads by clicking on their names on the left hand side of the edit window, and choose 'Realign Selection' from the 'Command' menu.

CRITICAL STEP
Stutter noise from replication slippage in SSRs causes divergent reads and low-quality base calls. In some cases, unambiguous resolution of these repeats may not be possible, and they should be annotated as unresolved in Step 71.

70 Remove any vector-sequence contamination at the ends of the clone. In the Gap5 edit window, use the 'Search' button to search the consensus sequence for the sequences at the cloning site of your vector. Trim away the vector sequence outside of the restriction sites used to generate your clone library (usually EcoRI, BamHI, or MboI).

71 Annotate any remaining ambiguities in the clone sequence (e.g., unresolved simple sequence repeats, where neither Illumina or nanopore reads are completely accurate) by compiling a feature table, which will be useful when finished clone sequences are submitted to GenBank.

TROUBLESHOOTING
Troubleshooting advice can be found in **Table 1**.
